# A comprehensive assessment of the cholinergic‐supporting and cognitive‐enhancing effects of *Rosa damascena* Mill. (Damask rose) essential oil on scopolamine‐induced amnestic rats

**DOI:** 10.1002/brb3.3507

**Published:** 2024-04-30

**Authors:** Kerem Teralı, Dilek Ozbeyli, Duygu Yiğit‐Hanoğlu, Kemal Hüsnü Can Başer, Göksel Şener, Asli Aykac

**Affiliations:** ^1^ Department of Medical Biochemistry, Faculty of Medicine Cyprus International University Nicosia Cyprus; ^2^ Department of Medical Services and Techniques, Vocational School of Health Services Marmara University Istanbul Turkey; ^3^ Department of Pharmacognosy, Faculty of Pharmacy Near East University Nicosia Cyprus; ^4^ Department of Pharmacology Fenerbahce University Istanbul Turkey; ^5^ Department of Biophysics Near East University Nicosia Cyprus

**Keywords:** Alzheimer's disease, cholinergic system, cognitive functions, scopolamine‐induced amnesia

## Abstract

**Introduction:**

Alzheimer's disease (AD) is a neurodegenerative condition characterized by gradual loss of cognitive abilities (dementia) and is a major public health problem. Here, we aimed at investigating the effects of *Rosa damascena* essential oil (RDEO) on learning and memory functions in a rat model of amnesia induced by scopolamine, as well as on changes in acetylcholinesterase (AChE) activity, M_1_ muscarinic acetylcholine receptor (mAChR) expression, and brain‐derived neurotrophic factor (BDNF) levels in the extracted brain tissues.

**Methods:**

The control, amnesia (scopolamine, 1 mg/kg/i.p.) and treatment (RDEO, 100 μL/kg/p.o. or galantamine, 1.5 mg/kg/i.p.) groups were subjected to Morris water maze and new object recognition tests. AChE activity was assayed by ELISA, and M_1_ mAChR and BDNF concentration changes were determined by western blotting. Also, using computational tools, human M_1_ mAChR was modeled in an active conformation, and the major components of RDEO were docked onto this receptor.

**Results:**

According to our behavioral tests, RDEO was able to mitigate the learning and memory impairments caused by scopolamine in vivo. Our in vitro assays showed that the observed positive effects correlated well with a decrease in AChE activity and an increase in M_1_ mAChR and BDNF levels in amnestic rat brains. We also demonstrated in an in silico setting that the major components of RDEO, specifically ‐citronellol, geraniol, and nerol, could be accommodated favorably within the allosteric binding pocket of active‐state human M_1_ mAChR and anchored here chiefly by hydrogen‐bonding and alkyl–π interactions.

**Conclusion:**

Our findings offer a solid experimental foundation for future RDEO‐based medicinal product development for patients suffering from AD.

## Graphical abstract

Finding effective preventative and therapeutic solutions for the management of Alzheimer's disease (AD) should be urgent and important for researchers given the world's increasingly aging population. Despite the tragic rise in patients with AD globally, there is presently no viable cure or means to arrest the disease. Acetylcholinesterase inhibitors, such as donepezil, rivastigmine, and galantamine, are used as part of the current treatment regimen but do not genuinely restore brain function in a clinical setting; instead, they just delay the advancement of AD. This places natural product research, which addresses various pathobiological processes tied to AD, at the center of anti‐AD drug discovery and development efforts. This is the first study to investigate the putative neuroprotective and pro‐cognitive activities of R. damascena essential oil against scopolamine‐induced amnesia of AD‐type in Wistar albino rats.

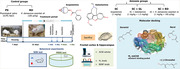

## 1 INTRODUCTION

It is widely established that an increase in average life expectancy is a positive indicator in terms of health. However, this increase is also responsible for the emergence of chronic diseases and dementia in various forms in the elderly. Patients’ quality of life is adversely affected due to neurodegenerative diseases, which are by impairments in memory and regression in learning ability, has become an increasing public health problem worldwide (Okano et al., 2000). Many anatomical regions, such as the medial temporal lobe and a variety of cortical and subcortical structures, are able to engage in learning and memory functions (Aggleton & Brown, 1999). These anatomical regions interact with diverse neurotransmitters that play positive or negative regulatory roles in the brain.

Acetylcholine (ACh) is an important neurotransmitter in both the central and peripheral nervous systems, providing communication between two nerve cells. One of the most important features of Alzheimer's disease (AD) is cholinergic hypofunction, which causes significant cholinergic neuronal abnormalities/losses with decreased choline acetyltransferase levels and accompanying reduced ACh synthesis (Francis et al., 1999). Through blocking the enzymatic hydrolysis of ACh by acetylcholinesterase (AChE), it is possible to enhance cholinergic function in AD by prolonging the release of ACh into the neuronal synaptic cleft (Amenta & Tayebati, 2008). Approved pharmacological treatments for AD include cholinergic agonists and AChE inhibitors (AChEIs) including rivastigmine, galantamine, and donepezil. Muscarinic ACh receptors (mAChRs), one of two main types of pharmacologically distinct ACh receptors that respond specifically to muscarine, also play an important role in AD pathology. ACh is released from vesicles in presynaptic neurons and binds to postsynaptic receptors. ACh, which moves by diffusion in the synaptic space, acts directly through G‐protein‐related secondary messengers by binding to mAChRs (Felder et al., 2000). mAChRs affect various biological pathways in the brain to govern crucial processes, such as neuronal excitability, synaptic plasticity, and feedback regulation of ACh release. Consequently, ACh and mAChRs are involved in the generation of higher‐order brain activities, such as learning and memory (Bradley et al., 2017; Ehlert et al., 1995; Navarria et al., 2015).

M_1_, the most abundant mAChR subtype in the brain with postsynaptic localization in the hippocampus, striatum, cerebral cortex, and sympathetic ganglia, governs neuronal cognition, memory, and movement signals. Because these receptors are known to play a key part in learning and memory, emotional and cognitive disorders are observed when M_1_ mAChR activity is blocked (Deiana et al., 2011; Klinkenberg et al., 2011; Schliebs & Arendt, 2011). Scopolamine is a nonselective mAChR antagonist that impacts learning acquisition and short‐term memory functions in both animals and humans (Wohleb et al., 2016). Scopolamine‐induced rodent models of amnesia are believed to be nondegenerative; therefore, scopolamine is frequently employed to establish experimental animal models in antiamnesic drug discovery research (Wang et al., 2010). On the other hand, there exist some experimental animal studies suggesting that scopolamine leads to a decrease in the number of neurons containing M_1_ receptors (Araujo et al., 2011; Jahanshahi et al., 2013). It is worth noting here that receptor tyrosine kinase B‐mediated brain‐derived neurotrophic factor (BDNF) signaling and M_1_ mAChR‐mediated ACh signaling share a common biological pathway involving protein kinase Cγ to potentiate ACh release and preserve presynaptic maintenance (Takamori, 2019; Tomàs et al., 2017). It has been proposed that neurotrophic factors, such as BDNF, may decelerate the course of neurodegeneration and serve as a viable option for AD treatments since the cognitive impairment in AD is caused by neurodegeneration (Gao et al., 2022).

The genus *Rosa* represents one of the most important genera of Rosaceae family and has 200 species and nearly 20,000 varieties (Rezvani‐Kamran et al., 2017). It has been suggested that the extracts of the plant, particularly those used in perfumery, are also potential sources of antioxidants that can be employed in food preservation (Verma et al., 2011). *Rosa damascena* (*R. damascene*) Mill. (Damask rose) is one of the most important fragrant rose species from which essential oils and high‐value products may be derived and widely used in traditional medicine to treat fevers, sore throats, and gastrointestinal symptoms (Baser et al., 2012; Mohammadpour et al., 2015). According to the literature, *R. damascena* possesses sedative, anti‐inflammatory, analgesic, and antioxidant properties (Akram et al., 2020; Boskabady et al., 2011; Hajhashemi et al., 2010). There is also substantial evidence that *R. damascena* extract significantly stimulates neurite growth and prevents amyloid‐beta (Aβ) fibrillation and accumulation in the brain (Awale et al., 2011). Different *R. damascena* extracts contain various compounds such as geraniol, citronellol, farnesol, nerol, linalool, citral, eugenol, phenethyl alcohol, myrcene, vitamin C, and bioflavonoids (Naquvi et al., 2014; Pellati et al., 2013). Flavonoid‐rich alcohol extracts of *R. damascena*, for example, are known to participate in many neuroprotective processes that support memory and other cognitive processes (Naquvi et al., 2014; Spencer, 2010).

To our knowledge, the present study is the first of its kind to explore, by behavioral experiments, the potential beneficial effects of *R. damascena* essential oil (RDEO) on memory and learning abilities impaired by scopolamine treatment in rats. It also investigates the underlying molecular mechanisms of cognitive (dys)function through assaying the enzymatic activity of AChE and identifying the amounts of M_1_ mAChR and BDNF expression in the harvested hippocampal and prefrontal cortical tissues. Finally, to predict whether RDEO possesses positive allosteric modulatory (PAM) properties, the major components of RDEO are docked in a computational setting onto active‐state human M_1_ mAChR.

## 2 MATERIALS AND METHODS

### 2.1 Animals and experimental design

**FIGURE 1:**
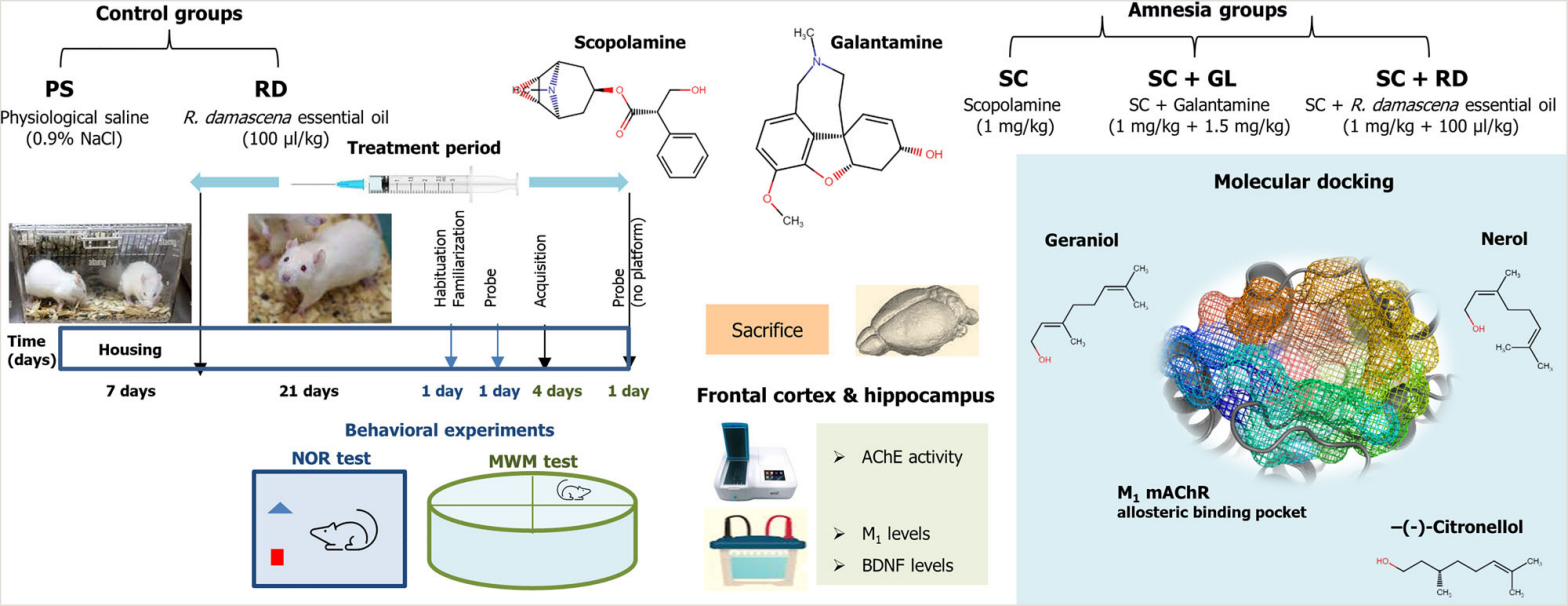
Workflow diagram of experimental design. AChE, acetylcholinesterase; BDNF, brain‐derived neurotrophic factor; GL, galantamine; M_1_, muscarinic acetylcholine receptor subtype M_1_; MWM, Morris water maze; NOR, novel object recognition; PS, physiological saline; RD, *Rosa damascena* essential oil; SC, scopolamine.

Male Wistar albino rats (8 weeks old and 220 ± 20 g body weight) were used in the study. The rats (*n* = 4) in cages received a standard diet with free access to drinking water. All rats were formed at random from rats that had been habituated to their home environment for 1 week, and their treatments were administered for 28 days according to the experimental protocol (Figure 1).

In determining the optimal dosage for our study, consideration was given to the range of doses employed by previous researchers in animal experiments involving orally administered (p.o.) *R. damascena* essential/volatile oil, typically ranging from 100 to 400 μL/kg (Hajhashemi et al., 2010; Latifi et al., 2015). To maintain consistency with the existing literature while conducting a preliminary investigation, the decision was made to deliberately opt for the smallest test dose within this range. Similarly, the selection of the scopolamine dose for our study was guided by a precedent set in the existing literature, where several researchers have consistently employed a dose of 1 mg/kg, administered intraperitoneally (i.p.), to induce amnesia in animal models (Hasanein & Mahtaj, 2015; Imam et al., 2016; Sajjad Haider et al., 2021).

The rats were divided into two control and three amnesia groups. The control group was given either physiological saline (untreated group designated as “PS”; rats injected with 0.9% NaCl) or RDEO (vehicle group designated as “RD”; rats administered with 100 μL/kg, p.o., RDEO) (Ramezani et al., 2008). To generate the experimental amnestic rat model, scopolamine was delivered (group designated as “SC”; rats administered with 1 mg/kg, i.p., scopolamine). In two other experimental models, amnesia was first induced by scopolamine and then treated with either galantamine (group designated as “SC+GL”; rats administered with 1.5 mg/kg, i.p., galantamine) or RDEO (group designated as “SC+RD”; rats administered with 100 μL/kg, p.o., RDEO) (Figure 1).

Throughout the study, the treatments given to the rodents were administrated at the same time (10:00 a.m.) every day.

### 2.2 Novel object recognition test

Each rat was given the novel object recognition (NOR) test consisting of the following stages: habituation/adaptation, familiarization, and testing. The NOR test was carried out in line with the previously established protocol and setup (Antunes & Biala, 2012). The rat was left alone in the test setting for 10 min on the first day to become acquainted to it. The rodent was placed in the setup on the second day after two identical objects were introduced in the test environment. The recordings were taken on the last day by placing the animal in the testing setup after one of the objects was altered.

### 2.3 Morris water maze test

A tank with opaque water and a submerged escape platform was employed for the test. To make the rats locate the platform, they were dropped into the tank at various starting points, and their learning was evaluated over the course of repeated trials (Aykac et al., 2022; Pandareesh et al., 2016; Vorhees & Williams, 2006). The platform was removed from the tank on the experiment's last day, and the animals’ orientation to the quadrant where the platform had previously been situated was used to assess spatial memory. The following parameters were analyzed using the Morris water maze (MWM) test: escape latency time (ELT) to discover the hidden platform, escape rate between the guardant, and time spent (TS) searching for the hidden platform in the target quadrant. Each was captured using a video camera mounted on the top of the test equipment.

### 2.4 Determination of protein expression and cholinesterase activity in dissected tissues

After the rats were sacrificed, the hippocampus and prefrontal cortex regions were dissected according to the coordinates and anatomical information from the Paxinos and Watson rat brain atlas (Paxinos & Watson, 2007) for BDNF and M_1_ mAChR immunoblot analyses and AChE activity assay. The tissues were homogenized in buffer (pH 7.4) by using a homogenizer, and, following centrifugation, total protein content in the cleared lysates was estimated by employing the Lowry method (Lowry et al., 1951). Next, the samples containing 100 μg of protein were loaded onto an SDS–polyacrylamide gel (12%), and the proteins were transferred from the gel onto a suitable membrane for antibody staining and detection. The membrane was first treated with primary antibody overnight, followed by secondary antibody for 1 h. All primary antibodies were used at a 1:100 dilution, except for β‐actin, which was used at a 1:200 dilution for normalization purposes. The Bio‐Rad Molecular Analyst software (free version, www.totallab.com) was implemented for the analyses. The activity of the ACh‐hydrolyzing enzyme AChE was measured spectrophotometrically at 420 nm (Synergy H1, BioTek Instruments, Inc.), based on the method of Sharma et al. (2008).

### 2.5 Homology modeling and molecular docking

The 3D conformers of PQCA (compound ID: 25229646), (−)‐citronellol (compound ID: 7793), geraniol (compound ID: 637566), and nerol (compound ID: 643820) in SDF format were retrieved from the PubChem open chemistry database (Kim et al., 2021). Given the absence of atomic coordinates for the active‐state M_1_ mAChR in the Protein Data Bank, the receptor was homology‐modeled by using the SWISS‐MODEL protein structure prediction server (Waterhouse et al., 2018). The amino acid sequence of human M_1_ mAChR (protein ID: P11229) was downloaded from the UniProt Knowledgebase (UniProt Consortium, 2021). The appropriate hydrogen coordinates and protonation states were assigned to the active M_1_ mAChR model via Protoss optimization (Bietz et al., 2014). The ligands were docked onto the receptor by using the JAMDA molecular docking tool that combines the TrixX docking algorithm (Henzler et al., 2014; Schellhammer & Rarey, 2007) with the JAMDA scoring function (Flachsenberg et al., 2020). The allosteric binding site of the active M_1_ mAChR model was defined by the pocket residues previously predicted by the DoGSiteScorer structure‐based method (Volkamer et al., 2010). Receptor–ligand docking was achieved with medium precision.

### 2.6 Statistical analysis

All data were analyzed and expressed as means SEM using the GraphPad Prism 6 program (GraphPad Software Inc.). Data from MWM trials were analyzed by repeated‐measures two‐way ANOVA. In the examination of other parameters, one‐way ANOVA followed by a Bonferroni *post hoc* test was utilized. *p* <.05 was deemed statistically significant for all analyses.

## 3 RESULTS

### 3.1 NOR test

**FIGURE 2:**
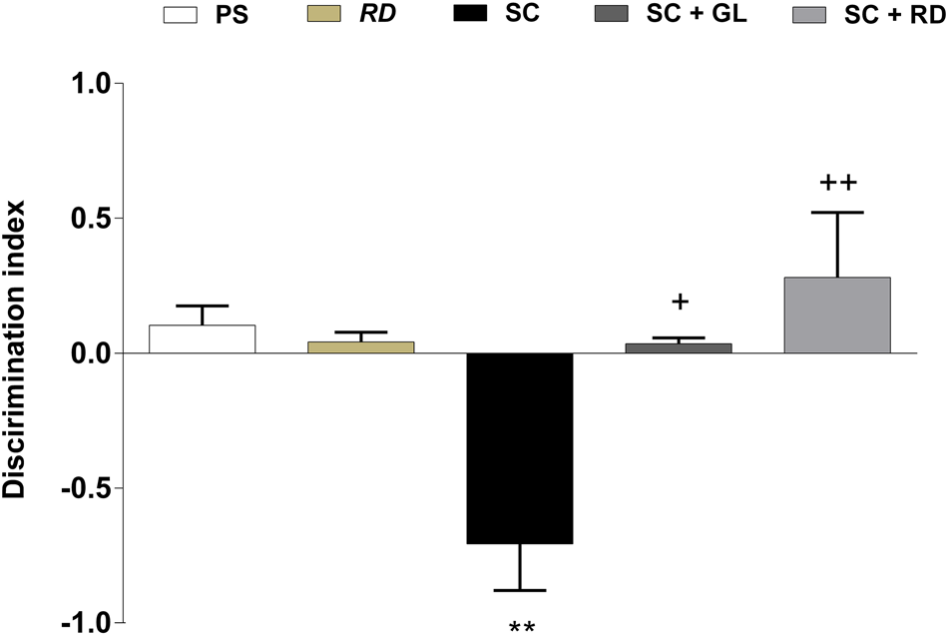
Discrimination index (ranging from −1 to +1) obtained from NOR test used to evaluate short‐term recognition memory. Six rats per group. ^*^ *p* <.05; ^**^ *p* <.01; ^***^ *p* <.001 significant as compared to the control. ^+^ *p* <.05; ^++^ *p* <.01; ^+++^ *p* <.001 significant as compared to the scopolamine‐induced amnesia group.

The NOR test was used to investigate the potential beneficial effects of galantamine and RDEO therapies on short‐term recognition memory impaired by scopolamine administration. The TS with the novel object and the TS with the familiar object in the amnesia group decreased as a result of scopolamine administration, which led to a fall in the discriminating index (*p* < .01; Figure 2). On the other hand, it was discovered that both galantamine and RDEO treatments given to the amnesia group increased the discriminating index by reducing the amount of TS exploring the novel object (*p* < .05−.01; Figure 2).

### 3.2 MWM test

**FIGURE 3:**
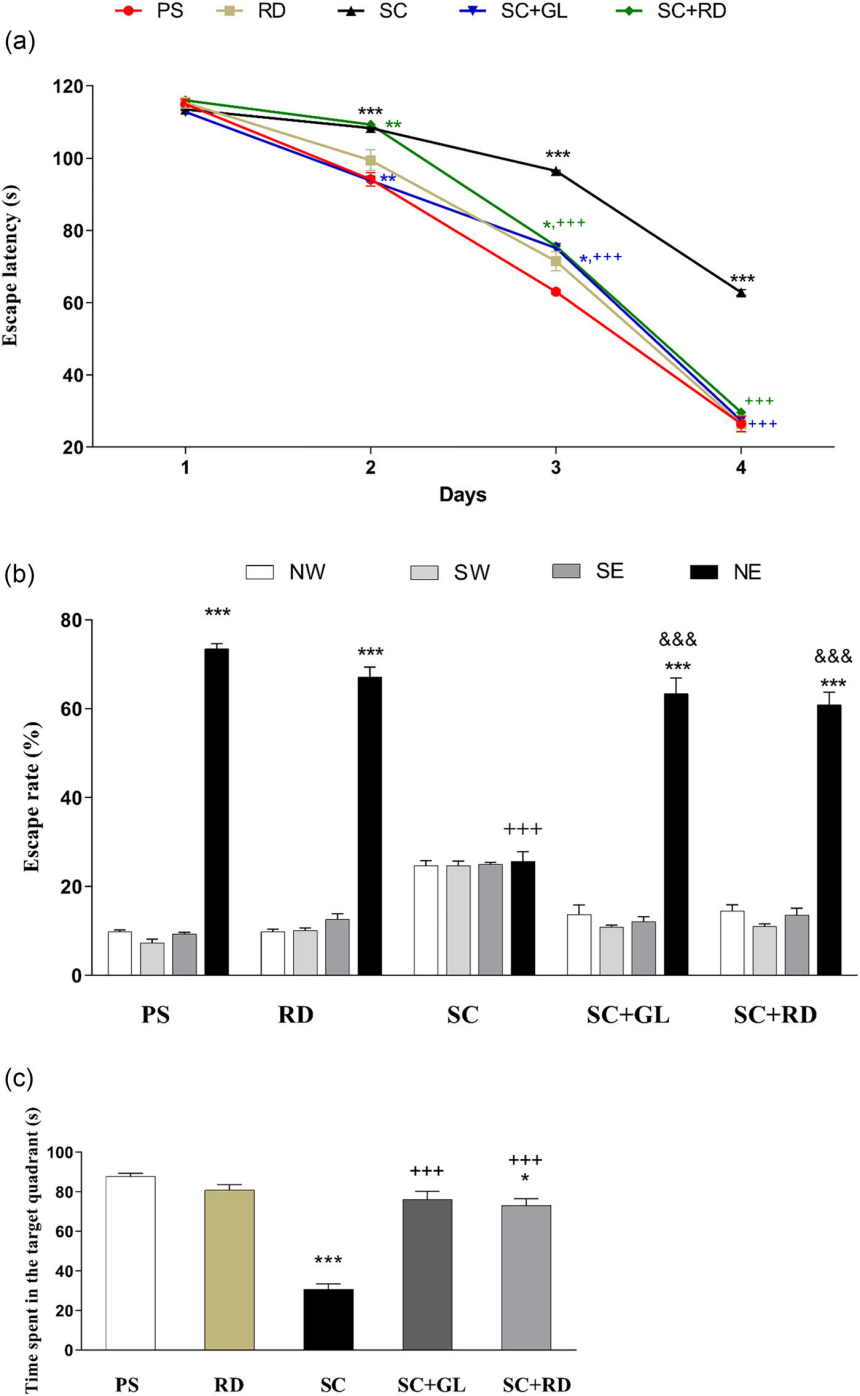
Effects of *Rosa damascena* essential oil on SC‐induced impairment of cognition, learning and recognition, and spatial memory function. (a) Escape latency time to find platform (s), (b) escape rate between quadrants (%), and (c) time spent in the target quadrant (s) in the Morris water maze. Six rats per group. ^*^ *p* <.05; ^**^ *p* <.01; ^***^ *p* <.001 significant for comparison of the time spent in the NE quadrant with that in the other quadrants (NW, SE, and SW) within each group. ^+^ *p* <.05; ^++^ *p* <.01; ^+++^ *p* <.001 significant for comparison of the time spent in the NE quadrant of the control group with the NE quadrant of other groups. ^&^ *p* <.05; ^&&^ *p* <.01; ^&&&^ *p* <.001 significant for comparison of the time spent in the NE quadrant of the SC group with the NE quadrant of other groups.

It was observed that the ELT of the rats during the MWM test to locate the platform in the PS and RD groups decreased significantly from the first day to the fourth day of the experiment (*p* <.001 in both groups). The change in ELT for the SC group was found to be 113.5 ± 11.7 s on the first day, and 62.8 ± 9.7 s on the fourth day. The effect of galantamine or RDEO treatment on ELT in the amnesia groups was 112.8 ± 9.7 s (SC+GL) and 116.0 ± 9.8 s (SC+RD) on the first day, and 27.3 ± 7.5 s (SC+GL) and 29.7 ± 6.6 s (SC+RD) on the fourth day (*p* <.001 in both groups; Figure 3a).

When the percentage distribution of the rats’ amount of TS in different quadrants (NE, northeast; NW, northwest; SE, southeast; SW, southwest) in the experimental groups on the fifth day was evaluated, it was determined that all groups, except the SC group, were found in the NE quadrant with a higher distribution ratio than in three other quadrants (Figure 3b). The proportion of total TS in the NE quadrant of the PS, RD, SC+GL, and SC+RD groups was higher than the SC group on the fifth day, as indicated by percentage distribution (*p* <.001).

TS in the target quadrant on the fifth day, which is one of the parameters reflecting the memory functions of the rats in the PS group, was found to be significantly longer than TS in the three other quadrants (87.8 ± 1.5; Figure 3c). When the platform recall behavior of the rats was examined, the total TS in the target quadrant of the rats in the RD group on the fifth day was 80.9 ± 2.7 s, and the TS in this quadrant was significantly longer than the TS in the other three quadrants. Although the TS in the target quadrant in the SC group was 30.8 ± 2.6 s, this time was shorter than the PS group (*p* <.001). The results show that rats in the SC+GL and SC+RD groups spent a significantly longer time (76.1 ± 4.1 s and 73.0 ± 3.4 s) in the target quadrant compared to the SC group (*p* <.001). In addition, in our MWM test results, it was seen that the increase in TS in the target quadrant detected on the fifth day in the SC+RD group almost approached the values of the PS group (*p* < .05). Day 5 of treatments significantly prevented the increase in scopolamine‐induced ELT and the reduction in TS in the target quadrant, indicating significant regression of scopolamine‐induced memory impairment.

### 3.3 Activity of AChE and immunoblotting of M_1_ mAChR and BDNF in dissected tissues

**FIGURE 4:**
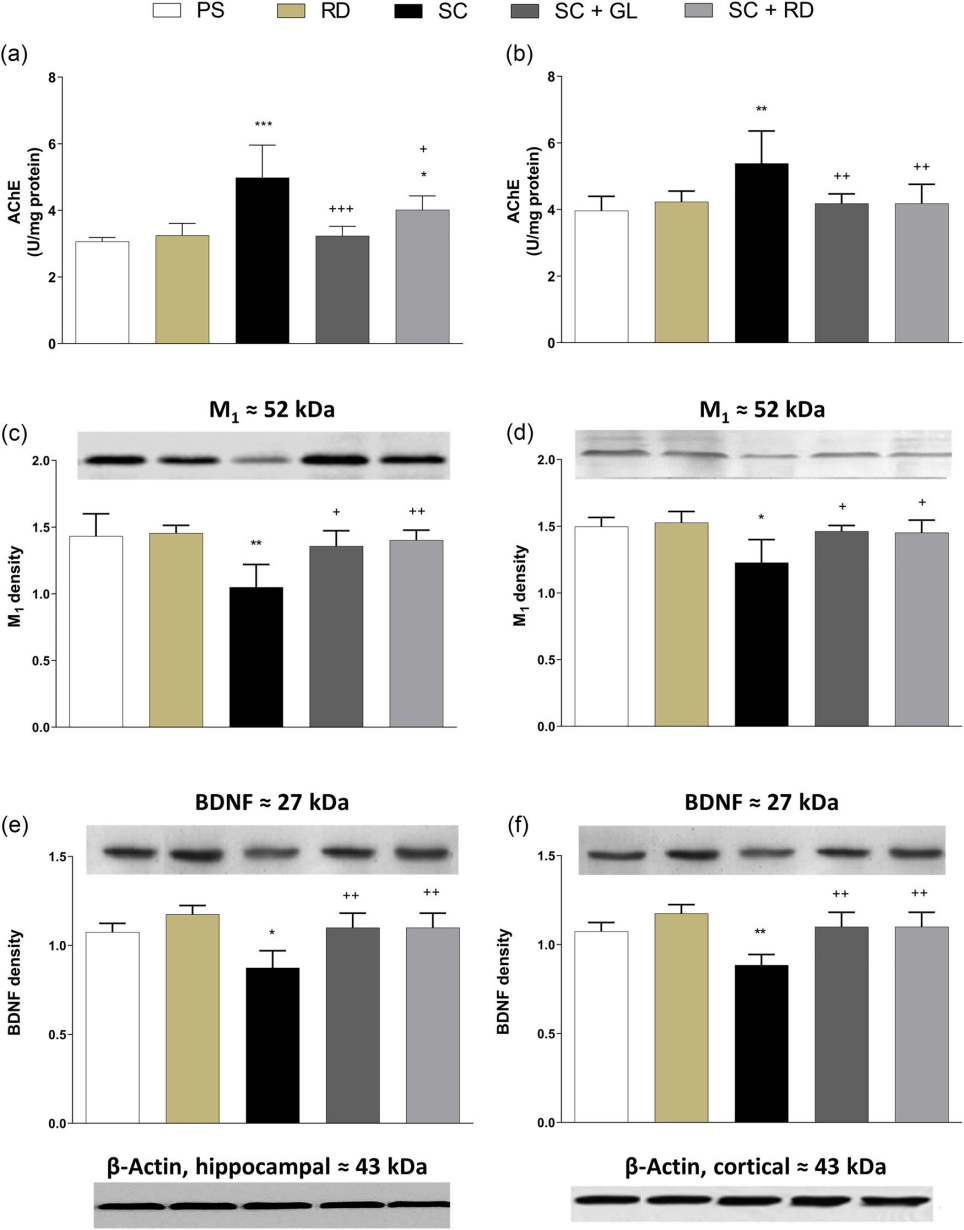
(a, b) Acetylcholinesterase activity, density of immunoblotting of (c, d) M_1_ expression levels and (d, e) BDNF levels of the hippocampus and frontal cortex regions of rats in SC‐induced AD rat model. AChE, acetylcholinesterase; BDNF, brain‐derived neurotrophic factor; GL, galantamine; M_1_, muscarinic acetylcholine receptor subtype M_1_; PS, physiological saline; RD, *Rosa damascena* essential oil; SC, scopolamine. Six rats per group. ^*^ *p* <.05; ^**^ *p* <.01; ^***^ *p* <.001 significant as compared to the control. ^+^ *p* <.05; ^++^ *p* <.01; ^+++^ *p* <.001 significant as compared to the scopolamine‐induced amnesia group.

While scopolamine applied for 28 days to induce amnesia caused a significant increase in AChE activity in the hippocampal and cortical regions (*p* < .001 and *p* < .01, respectively) of the SC group compared to the untreated control group (PS), galantamine and RDEO treatments applied to the amnesia group significantly decreased the activity of this enzyme (*p* < .001 and *p* < .01 in the hippocampus, respectively; *p* < .05 and *p* < .01 in the cortex, respectively) (Figure 4a,b).

The expression levels of M_1_ mAChR significantly decreased with the administration of scopolamine compared to the PS group both in the hippocampus (*p* < .01, Figure 4c) and in the cortex (*p* < .05) (Figure 4d). The expression levels of M_1_ mAChR in the SC+GL and SC+RD amnesia groups significantly increased compared to the SC group both in the hippocampus (*p* < .05 and *p* < .01, respectively) and in the cortex (*p* < .05 in both groups).

Since neurotrophic factors are known to be associated with neurogenesis, neuroplasticity, learning, and memory, we investigated whether RD could modulate BDNF expression. The expression levels of BDNF significantly decreased with the administration of scopolamine compared to the PS group in the hippocampal and cortical brain areas (*p* < .05, Figure 4e and *p* < .01, Figure 4f, respectively). The expression levels of BDNF in the SC+GL and SC+RD amnesia groups significantly increased compared to the SC group both in the hippocampus (*p* < .01 in both groups) and in the cortex (*p* < .01 in both groups). Our data showed that RD successfully restored low levels of BDNF in a rat model of scopolamine‐induced amnesia.

### 3.4 In silico studies

**FIGURE 5:**
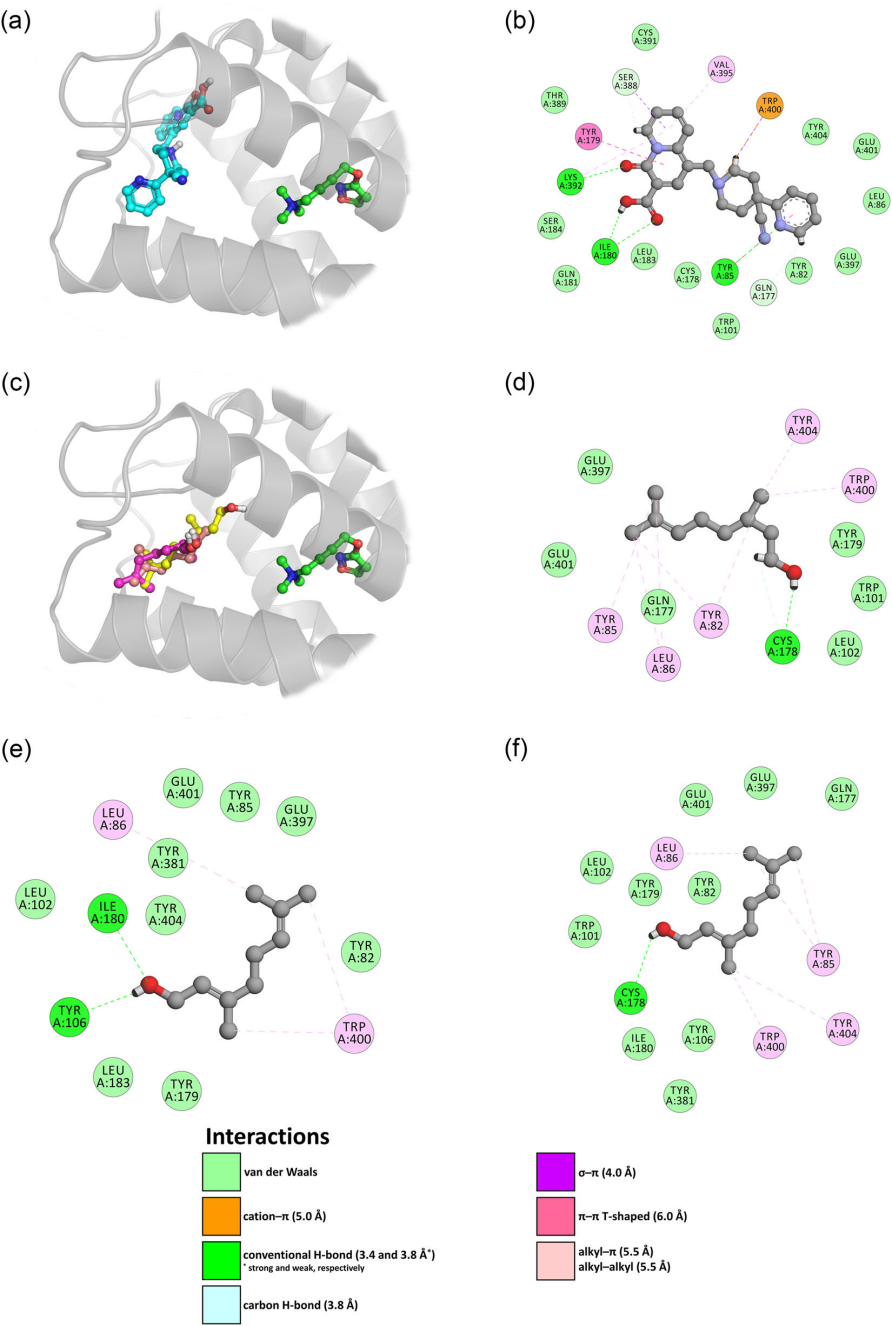
Results of protein–ligand docking and interaction profiling. (a) Top‐scoring binding pose of PQCA (cyan sticks), a highly selective positive allosteric modulator of M_1_ mAChR, shown in the allosteric binding pocket of the active‐state human M_1_ mAChR homology model (gray ribbons). Iperoxo, a potent superagonist of mAChR, is also shown as green sticks in the orthosteric binding site of the modeled receptor. Nonpolar hydrogen atoms are hidden for clarity. (b) Favorable noncovalent interactions stabilizing the binding of PQCA in the receptor's allosteric binding pocket. Only interacting hydrogen atoms are shown for clarity. (c) Top‐scoring binding poses of (–)‐citronellol (magenta sticks), geraniol (yellow sticks), and nerol (orangey‐pink sticks), the major components of RDEO, shown in the allosteric binding pocket of the active‐state human M_1_ mAChR homology model (gray ribbons). Iperoxo, a potent superagonist of mAChR, is also shown as green sticks in the orthosteric binding site of the modeled receptor. Nonpolar hydrogen atoms are hidden for clarity. Calculated favorable noncovalent interactions stabilizing the binding of (d) (–)‐citronellol, (e) geraniol, and (f) nerol in the receptor's allosteric binding pocket. Only interacting hydrogen atoms are shown for clarity. The images (a) and (c) were rendered using the PyMOL Molecular Graphics System, v1.8 (Schrödinger LLC). The images (b) and (d–f) were rendered using Discovery Studio Visualizer, v16.1.0 (Dassault Systèmes BIOVIA Corp.).

The results of RDEO content analysis (high/low content of organic compounds) revealed that citronellol (29.5%), geraniol (20.2%), nonadecane (16.5%), nerol (9.6%), heneicosane (5.5%), nonadecene (3.2%), heptadecane (2.6%), and methyl eugenol (1.6%) constituted most of the components in the oil (Table S1). In view of the partial lack of experimental data on conformationally diverse M_1_ mAChR structures, a homology model of the active human M_1_ mAChR was built based on the template crystal structure of the active human M_2_ mAChR bound to the orthosteric agonist iperoxo and the PAM LY2119620 (PDB ID: 4MQT) (Kruse et al., 2013). Human M_2_ mAChR shares a sequence identity of 65.44% with human M_1_ mAChR over a length of 412 amino acid residues, indicating to the correct fold of the resulting homology model. The allosteric binding site of human M_1_ mAChR is lined by the residues Tyr82, Tyr85, Leu86, Gly89, His90, Trp101, Leu102, Tyr106, Thr172, Gln177, Cys178, Tyr179, Ile180, Gln181, Leu183, Ser184, Thr189, Tyr381, Val385, Ser388, Thr389, Cys391, Lys392, Val395, Glu397, Trp400, Glu401, and Tyr404. Molecular docking with the known PAM PQCA showed that the ligand could be housed well in the allosteric pocket of the receptor (Figure 5a). The highest‐scoring (JAMDA score: −2.74978) binding pose of PQCA was able to establish hydrogen‐bonding interactions with the allosteric pocket residues of M_1_ mAChR through its oxo, carboxyl, and pyridyl groups. PQCA appeared to be further anchored within the allosteric pocket of M_1_ mAChR by π‐effects, namely, cation–π, σ–π, π–π, and alkyl–π interactions (Figure 5b). Cross‐docking calculations demonstrated that the major components of RDEO could also be accommodated in the allosteric pocket of M_1_ mAChR (Figure 5c), albeit with lower JAMDA scores. (−)‐Citronellol (JAMDA score: −1.82336) was able to form a conventional H‐bond with the main chain of Cys178 through its hydroxyl group and also multiple alkyl–π interactions with the surrounding aromatic residues (Tyr82, Tyr85, Trp400, and Tyr404) (Figure 5d). Geraniol (JAMDA score: −1.95605) could engage in conventional H‐bonds with the side chain of Tyr106 and the main chain of Ile180 through its hydroxyl group as well as in alkyl–π interactions with the aromatic ring of Trp400 (Figure 5e). Nerol (JAMDA score: −1.80553) was able to establish a conventional H‐bond with the main chain of Cys178 through its hydroxyl moiety and also multiple alkyl–π interactions with the nearby aromatic residues (Tyr85, Trp400, and Tyr404) (Figure 5f). In each case, Leu86 appeared to further stabilize the binding of the ligand at the allosteric binding site of the receptor by forming alkyl–alkyl interactions with the ligand through its branched hydrocarbon side chain.

## 4 DISCUSSION

In the present study, we observed the effects of RDEO on learning and memory functions in the rat model of scopolamine‐induced amnesia, as well as the changes in AChE activity, M_1_ mAChR expression, and BDNF levels. RDEO demonstrates positive effects on cognitive functions, as evidenced by behavioral tests, and is linked to biochemical changes, including a reduction in AChE activity and an elevation in M_1_ mAChR and BDNF levels in amnestic rat brains. Computational modeling provides additional clarity on the potential molecular interactions between RDEO components and the human M_1_ mAChR, presenting insightful findings.

Despite being elegant and robust, previous studies on *R. damascena* are limited in number and scope. For example, in a rat model of Aβ_1−42_‐induced AD, Esfandiary et al. (2014) demonstrated that oral administration of dried, standardized *R. damascena* extract in an aqueous medium stimulated both adult neurogenesis and synaptic plasticity and also reversed the Aβ Aβ_1−42_‐associated memory deficits. Also, in an *ex vivo* model of AD, Awale et al. (2011) showed that the chloroform extract of *R. damascena* promoted neurite outgrowth and inhibited the Aβ_25−35_‐induced atrophy and cell death in rat cortical neurons. In another study by Zhu et al. (2017), rose essential oil extracted from *R. setate* × *R. rugosa* (Chinese Kushui rose) was found to suppress AD‐like symptoms, such as paralysis, and reduce Aβ_1−42_ oligomers via the SKN‐1 signaling pathway (which is homologous to the Nrf2 signaling pathway in mammals) in a transgenic *Caenorhabditis elegans* model of AD. In light of the scarcity of research on this particular topic, our study is the first multiparameter investigation to consider the cholinergic facilitatory and cognitive‐promoting impacts of RDEO in a rat model of AD‐type amnesia generated by intraperitoneal injection of scopolamine.

The NOR test, which provides a fast object–location–context recognition memory performance evaluation for rodents, was utilized to assess memory dysfunction caused by scopolamine delivered i.p. to rats at a dose of 1 mg/kg. Rats given scopolamine were unable to differentiate between unfamiliar and familiar items, indicating that recognition was significantly impaired in this group of animals. However, when rats were given RDEO, the scopolamine‐induced decline in cognitive functions was reversed, and memory performance improved. The MWM test results showed that administering scopolamine to rats significantly lowered the ELT to discover the hidden platform and TS searching for the hidden platform in the target quadrant, both of which are cognitive‐behavioral markers of rodent learning skills and memory retrieval. However, RDEO treatment was discovered to improve scopolamine‐induced adverse effects on these parameters, implying to the modulatory role of this natural product in learning and memory performance.

Sustaining ACh synthesis and release in the brain is essential for the cognitive functions of this organ since the cholinergic system plays a significant role in learning and memory (Blake et al., 2014). The activity of AChE hydrolyzes the naturally occurring neurotransmitter ACh to acetate and choline at synaptic sites, which impedes or halts cholinergic neurotransmission (Ballard et al., 2005). Therefore, a modulator stimulating the action of AChE on ACh should ideally impair learning and memory through decreasing ACh levels in brain regions underlying cognitive function (such as the hippocampus and the neocortex), as scopolamine did in our amnesia groups. Contrarily, both the standard drug galantamine and RDEO significantly inhibited the ACh‐hydrolyzing activity of AChE in the current study, enabling the rats to preserve their memories of the tasks they learned during the behavioral experiments. Our findings agree with previous reports presenting evidence for the AChE inhibitory activity of rose (*R. damascena*) essential oil (Senol et al., 2011a; Senol et al., 2011b).

Cholinergic neurotransmission at M_1_ mAChR is known to be implicated in higher brain functions. Indeed, M_1_ mAChR has long been recognized as a prospective therapeutic target for improving cognitive deficits in AD because of its critical involvement in learning and memory processes (Scarpa et al., 2020). Data from postmortem studies suggest that Aβ induces G‐protein cleavage by antagonizing M_1_ mAChR function in the hippocampus, the brain region most affected by Aβ (Nathan et al., 2013). According to the findings of a study involving rodent AD models, depletion of M_1_ mAChR stimulates amyloid precursor protein (APP) processing in neurons and promotes brain Aβ plaque pathology (Davis et al., 2010). Another mechanism linking M_1_ mAChR to AD is the receptor's therapeutic effect on impaired cerebral blood flow in AD patients (Jiang et al., 2014). Jahanshahi et al. (2013) showed immunohistochemically that scopolamine treatment caused a significant reduction in the number of neurons containing M_1_ receptor in the hippocampal area in rats. Similar research on dogs has shown that scopolamine therapy decreases the quantity of mAChR‐containing neurons in sections of the parietal, frontal, and entorhinal cortex (Araujo et al., 2011). We discovered a drop in M_1_ mAChR expression upon scopolamine treatment in our study, which could be attributed to a loss of neurons with M_1_ receptors. On the other hand, the administration of galantamine and RDEO in the current research markedly boosted M_1_ expression.

Previous attempts to discover highly selective orthosteric agonists of M_1_ mAChR have failed despite recent developments and advances in medicinal chemistry due to the highly conserved nature of the orthosteric ACh‐binding site. To address this problem, several research groups have switched to developing molecules that can occupy allosteric binding sites on mAChRs, which are structurally separate from the orthosteric binding site and may be less conserved among receptor subtypes. To date, multiple subtype‐selective PAMs of the M_1_ receptor have been designed (Johnson et al., 2022). They prevent activation of other mAChR subtypes by targeting a preformed outer binding pocket on M_1_ mAChR. Benzyl quinolone carboxylic acid (BQCA), Merck's first prototypical M_1_ PAM, is a highly selective M_1_ mAChR activator causing no potentiation (up to 100 μM) of any other subtype. BCQA has been shown to attenuate scopolamine‐induced memory impairments in contextual fear conditioning tests, improve discriminating reversal learning in a transgenic mouse model of AD, control amyloidogenic APP processing in vitro, and reverse amphetamine‐induced hyperlocomotion, an in vivo antipsychotic efficacy testing (Conn et al., 2009; Ma et al., 2009). The subsequent and more potent M_1_ PAM PQCA (a quinolizidinone) has been demonstrated to reduce scopolamine‐induced deficits in NOR tests in rats, as well as in object retrieval and spatial self‐ordered search tests in rhesus and cynomolgus macaques, respectively (Uslaner et al., 2013). Here, in a computational environment, we showed that the major components of RDEO, namely, (−)‐citronellol, geraniol, and nerol, could be housed well within the allosteric binding pocket of the active‐state human M_1_ mAChR model and bound here mainly by hydrogen‐bonding and alkyl–π interactions.

Although the causes of AD and the progression of the disease are not fully understood, it is increasingly recognized that the chronic inflammatory state of the brain, or neuroinflammation, plays a very important role in these processes. There is a substantial body of data linking BDNF to neuronal plasticity, repair, and survival, as well as to neurotransmitter release, and patients with neurodegenerative diseases frequently have lower BDNF levels in their brain (Lima Giacobbo et al., 2019). As persistent exposure to proinflammatory cytokines is known to influence multiple BDNF‐related signaling pathways, a recent hypothesis postulates that these aberrant BDNF levels may be caused by neuroinflammatory changes in certain neurodegenerative diseases. Indeed, proinflammatory cytokines, particularly IL‐1β, have been shown to downregulate BDNF expression in cognition‐related brain regions such as the hippocampus (Lynch, 2015; Tong et al., 2012). In the present study, we demonstrated that RDEO treatment (in a similar manner to galantamine treatment) significantly antagonized scopolamine‐induced reduction in hippocampal and cortical BDNF concentrations. This allowed us to make promising projections on the correlation between elevated BDNF levels and the operational status of molecular and cellular mechanisms underlying learning and memory in rats.

We are aware that our study has several limitations, including the difficulty of both examining mood and behavior in experimental animals and translating animal data to human disease. Additionally, further study is required to explore the possible involvement of neurotransmitters, receptors, and channels other than those belonging to the cholinergic system in the cognitive‐enhancing activity of RDEO. However, it should be underlined that any therapy that alleviates symptoms, raises quality of life, slows or halts neurodegeneration, and modifies total disease costs represents a substantial advancement in the fight to find a solution for AD.

## 5 CONCLUSION

AD is the most common neurodegenerative disorder, yet treatment options are limited and generally ineffective. In this respect, multitargeting natural products, which can combine broad antioxidant and anti‐inflammatory mechanisms with particular receptor and/or enzyme‐mediated actions, continue to gain popularity and general acceptance as new therapeutic discoveries aiming at neuroprotection, neuroregeneration, neuroplasticity, and cognition. Our study on the effects of RDEO in a rat model of scopolamine‐induced amnesia reveals promising results. RDEO demonstrates a positive impact on learning and memory functions, supported by behavioral tests, and is associated with biochemical changes such as decreased AChE activity and increased levels of M_1_ mAChR and BDNF in amnestic rat brains. Computational modeling further elucidates the potential molecular interactions between RDEO components and the human M_1_ mAChR, providing valuable insights. Overall, our findings provide a robust experimental foundation for future endeavors in RDEO‐based medicinal product development, offering a potential avenue for novel therapeutic interventions aimed at ameliorating cognitive decline in patients suffering from AD. The multifaceted approach, incorporating behavioral, biochemical, and computational analyses, contributes to a comprehensive understanding of the therapeutic potential of RDEO, marking a significant advancement in the quest for effective treatments for AD and related cognitive disorders.

## AUTHOR CONTRIBUTIONS

**Dilek Ozbeyli**: Study conception and design; data collection; analysis and interpretation of results; draft manuscript preparation;. **Göksel Şener**: Study conception and design. **Asli Aykac**: Study conception and design; data collection; analysis and interpretation of results; draft manuscript preparation. **Kerem Teralı**: data collection; analysis and interpretation of results; draft manuscript preparation. **Duygu Yiğit‐Hanoğlu**: data collection. **Kemal Hüsnü Can Başer**: Analysis and interpretation of results.

## CONFLICT OF INTEREST STATEMENT

The authors declare that they have no known competing financial interests or personal relationships that could have appeared to influence the work reported in this paper.

## FUNDING INFORMATION

The authors received no financial support or any sort of grant for this research.

### PEER REVIEW

The peer review history for this article is available at https://publons.com/publon/10.1002/brb3.3507

## Supporting information

Supporting Information

## Data Availability

The data that support the findings of this study are available upon reasonable request from the authors.

## References

[brb33507-bib-0001] Aggleton, J. P. , & Brown, M. W. (1999). Episodic memory, amnesia, and the hippocampal‐anterior thalamic axis. Behavioral and Brain Sciences, 22(3), 425–444. 10.1017/S0140525X99002034 11301518

[brb33507-bib-0002] Akram, M. , Riaz, M. , Munir, N. , Akhter, N. , Zafar, S. , Jabeen, F. , Ali Shariati, M. , Akhtar, N. , Riaz, Z. , Altaf, S. H. , Daniyal, M. , Zahid, R. , & Said Khan, F. (2020). Chemical constituents, experimental and clinical pharmacology of *Rosa damascena*: A literature review. Journal of Pharmacy and Pharmacology, 72(2), 161–174. 10.1111/jphp.13185 31709541

[brb33507-bib-0003] Amenta, F. , & Tayebati, S. (2008). Pathways of acetylcholine synthesis, transport and release as targets for treatment of adult‐onset cognitive dysfunction. Current Medicinal Chemistry, 15(5), 488–498. 10.2174/092986708783503203 18289004

[brb33507-bib-0004] Antunes, M. , & Biala, G. (2012). The novel object recognition memory: Neurobiology, test procedure, and its modifications. Cognitive Processing, 13(2), 93–110. 10.1007/s10339-011-0430-z 22160349 PMC3332351

[brb33507-bib-0005] Araujo, J. A. , Nobrega, J. N. , Raymond, R. , & Milgram, N. W. (2011). Aged dogs demonstrate both increased sensitivity to scopolamine impairment and decreased muscarinic receptor density. Pharmacology Biochemistry and Behavior, 98(2), 203–209. 10.1016/j.pbb.2011.01.005 21238475

[brb33507-bib-0006] Awale, S. , Tohda, C. , Tezuka, Y. , Miyazaki, M. , & Kadota, S. (2011). Protective effects of *Rosa damascena* and its active constituent on A {beta} (2535)induced neuritic atrophy. Evidence‐Based Complementary and Alternative Medicine, 2011, 131042. 10.1093/ecam/nep149 19789212 PMC3162985

[brb33507-bib-0007] Aykac, A. , Teralı, K. , Özbeyli, D. , Ede, S. , Albayrak, Ö. , Başer, K. H. C. , & Şener, G. (2022). A multi‐parameter evaluation of the neuroprotective and cognitive‐enhancing effects of *Origanum onites* L. (Turkish Oregano) essential oil on scopolamine‐induced amnestic rats. Metabolic Brain Disease, 37(4), 1041–1055. 10.1007/s11011-022-00933-6 35201555

[brb33507-bib-0008] Ballard, C. , Greig, N. , Guillozet‐Bongaarts, A. , Enz, A. , & Darvesh, S. (2005). Cholinesterases: Roles in the brain during health and disease. Current Alzheimer Research, 2(3), 307–318. 10.2174/1567205054367838 15974896

[brb33507-bib-0009] Baser, K. H. C. , Altintas, A. , & Kurkcuoglu, M. (2012). Turkish Rose: A review of the history, ethnobotany and modern uses of rose petals, rose oil, rose water and other rose products. Herbal Gram, 96, 40–53.

[brb33507-bib-0010] Bietz, S. , Urbaczek, S. , Schulz, B. , & Rarey, M. (2014). Protoss: A holistic approach to predict tautomers and protonation states in protein‐ligand complexes. Journal of Cheminformatics, 6(6), 12. 10.1186/1758-2946-6-12 24694216 PMC4019353

[brb33507-bib-0011] Blake, M. G. , Krawczyk, M. C. , Baratti, C. M. , & Boccia, M. M. (2014). Neuropharmacology of memory consolidation and reconsolidation: Insights on central cholinergic mechanisms. Journal of Physiology, Paris, 108(4–6), 286–291. 10.1016/j.jphysparis.2014.04.005 24819880

[brb33507-bib-0012] Boskabady, M. H. , Shafei, M. N. , Saberi, Z. , & Amini, S. (2011). Pharmacological effects of *Rosa damascena* . Iranian Journal of Basic Medical Sciences, 14, 295–307. 23493250 PMC3586833

[brb33507-bib-0013] Bradley, S. J. , Bourgognon, J.‐M. , Sanger, H. E. , Verity, N. , Mogg, A. J. , White, D. J. , Butcher, A. J. , Moreno, J. A. , Molloy, C. , Macedo‐Hatch, T. , Edwards, J. M. , Wess, J. , Pawlak, R. , Read, D. J. , Sexton, P. M. , Broad, L. M. , Steinert, J. R. , Mallucci, G. R. , Christopoulos, A. , … Tobin, A. B. (2017). M1 muscarinic allosteric modulators slow prion neurodegeneration and restore memory loss. Journal of Clinical Investigation, 127(2), 487–499. 10.1172/JCI87526 27991860 PMC5272187

[brb33507-bib-0014] Conn, P. J. , Jones, C. K. , & Lindsley, C. W. (2009). Subtype‐selective allosteric modulators of muscarinic receptors for the treatment of CNS disorders. Trends in Pharmacological Sciences, 30(3), 148–155. 10.1016/j.tips.2008.12.002 19201489 PMC2907736

[brb33507-bib-0015] Davis, A. A. , Fritz, J. J. , Wess, J. , Lah, J. J. , & Levey, A. I. (2010). Deletion of M1 muscarinic acetylcholine receptors increases amyloid pathology in vitro and in vivo. Journal of Neuroscience, 30, 4190–4196. 10.1523/JNEUROSCI.6393-09.2010 20335454 PMC2855655

[brb33507-bib-0016] Deiana, S. , Platt, B. , & Riedel, G. (2011). The cholinergic system and spatial learning. Behavioural Brain Research, 221(2), 389–411. 10.1016/j.bbr.2010.11.036 21108971

[brb33507-bib-0017] Ehlert, F. J. , Roeske, W. R. , & Yamamura, H. I. (1995). Molecular biology, pharmacology, and brain distribution of subtypes of the muscarinic receptor. In F. E. Bloom , & D. J. Kupfer (Eds). Psychopharmacology: The fourth generation of progress (pp. 111–124). Raven Press.

[brb33507-bib-0018] Esfandiary, E. , Karimipour, M. , Mardani, M. , Alaei, H. , Ghannadian, M. , Kazemi, M. , Mohammadnejad, D. , Hosseini, N. , & Esmaeili, A. (2014). Novel effects of *Rosa damascena* extract on memory and neurogenesis in a rat model of Alzheimer's disease. Journal of Neuroscience Research, 92(4), 517–530. 10.1002/jnr.23319 24395280

[brb33507-bib-0019] Felder, C. C. , Bymaster, F. P. , Ward, J. , & Delapp, N. (2000). Therapeutic opportunities for muscarinic receptors in the central nervous system. Journal of Medicinal Chemistry, 43(23), 4333–4353. 10.1021/jm990607u 11087557

[brb33507-bib-0020] Flachsenberg, F. , Meyder, A. , Sommer, K. , Penner, P. , & Rarey, M. (2020). Consistent scheme for gradient‐based optimization of protein‐ligand poses. Journal of Chemical Information and Modeling, 60(12), 6502–6522. 10.1021/acs.jcim.0c01095 33258376

[brb33507-bib-0021] Francis, P. T. , Palmer, A. M. , Snape, M. , & Wilcock, G. K. (1999). The cholinergic hypothesis of Alzheimer's disease: A review of progress. Journal of Neurology, Neurosurgery, and Psychiatry, 66, 137–147. 10.1136/jnnp.66.2.137 10071091 PMC1736202

[brb33507-bib-0022] Gao, L. , Zhang, Y. , Sterling, K. , & Song, W. (2022). Brain‐derived neurotrophic factor in Alzheimer's disease and its pharmaceutical potential. Translational Neurodegeneration, 11, 4. 10.1186/s40035-022-00279-0 35090576 PMC8796548

[brb33507-bib-0023] Hajhashemi, V. , Ghannadi, A. , & Hajiloo, M. (2010). Analgesic and anti‐inflammatory effects of *Rosa damascena* hydroalcoholic extract and its essential oil in animal models. Iranian Journal of Pharmaceutical Research, 9(2), 163–168. 24363723 PMC3862064

[brb33507-bib-0024] Hasanein, P. , & Mahtaj, A. K. (2015). Ameliorative effect of rosmarinic acid on scopolamine‐induced memory impairment in rats. Neuroscience Letters, 585, 23–27. 10.1016/j.neulet.2014.11.027 25445372

[brb33507-bib-0025] Henzler, A. M. , Urbaczek, S. , Hilbig, M. , & Rarey, M. (2014). An integrated approach to knowledge‐driven structure‐based virtual screening. Journal of Computer‐Aided Molecular Design, 28(9), 927–939. 10.1007/s10822-014-9769-4 24993405

[brb33507-bib-0026] Imam, A. , Ajao, M. S. , Ajibola, M. I. , Amin, A. , Abdulmajeed, W. I. , Lawal, A. Z. , Alli‐Oluwafuyi, A. , Akinola, O. B. , Oyewopo, A. O. , Olajide, O. J. , & Adana, M. Y. (2016). Black seed oil ameliorated scopolamine‐induced memory dysfunction and cortico‐hippocampal neural alterations in male Wistar rats. Bulletin of Faculty of Pharmacy, Cairo University, 54(1), 49–57.

[brb33507-bib-0027] Jahanshahi, M. , Nickmahzar, E. G. , Seif‐Hoseini, S. , Babakordi, F. , & Moharreri, A. (2013). Scopolamine reduces the density of M1 muscarinic neurons in rats' hippocampus. International Journal of Morphology, 31(4), 1227–1232. 10.4067/S0717-95022013000400014

[brb33507-bib-0028] Jiang, S. , Li, Y. , Zhang, C. , Zhao, Y. , Bu, G. , Xu, H. , & Zhang, Y.‐W. (2014). M1 muscarinic acetylcholine receptor in Alzheimer's disease. Neuroscience Bulletin, 30(2), 295–307. 10.1007/s12264-013-1406-z 24590577 PMC5562652

[brb33507-bib-0029] Johnson, C. R. , Kangas, B. D. , Jutkiewicz, E. M. , Bergman, J. , & Coop, A. (2022). Drug design targeting the muscarinic receptors and the implications in central nervous system disorders. Biomedicines, 10(2), 398. 10.3390/biomedicines10020398 35203607 PMC8962391

[brb33507-bib-0030] Kim, S. , Chen, J. , Cheng, T. , Gindulyte, A. , He, J. , He, S. , Li, Q. , Shoemaker, B. A. , Thiessen, P. A. , Yu, B. , Zaslavsky, L. , Zhang, J. , & Bolton, E. E. (2021). PubChem in 2021: New data content and improved web interfaces. Nucleic Acids Research, 49(D1), D1388–D1395. 10.1093/nar/gkaa971 33151290 PMC7778930

[brb33507-bib-0031] Klinkenberg, I. , Sambeth, A. , & Blokland, A. (2011). Acetylcholine and attention. Behavioural Brain Research, 221(2), 430–442. 10.1016/j.bbr.2010.11.033 21108972

[brb33507-bib-0032] Kruse, A. C. , Ring, A. M. , Manglik, A. , Hu, J. , Hu, K. , Eitel, K. , Hübner, H. , Pardon, E. , Valant, C. , Sexton, P. M. , Christopoulos, A. , Felder, C. C. , Gmeiner, P. , Steyaert, J. , Weis, W. I. , Garcia, K. C. , Wess, J. , & Kobilka, B. K. (2013). Activation and allosteric modulation of a muscarinic acetylcholine receptor. Nature, 504(7478), 101–106. 10.1038/nature12735 24256733 PMC4020789

[brb33507-bib-0033] Latifi, G. , Ghannadi, A. , & Minaiyan, M. (2015). Anti‐inflammatory effect of volatile oil and hydroalcoholic extract of *Rosa damascena* Mill. on acetic acid‐induced colitis in rats. Research in Pharmaceutical Sciences, 10(6), 514–522. 26779271 PMC4698862

[brb33507-bib-0034] Lima Giacobbo, B. , Doorduin, J. , Klein, H. C. , Dierckx, R. A. J. O. , Bromberg, E. , & De Vries, E. F. J. (2019). Brain‐derived neurotrophic factor in brain disorders: Focus on neuroinflammation. Molecular Neurobiology, 56(5), 3295–3312. 10.1007/s12035-018-1283-6 30117106 PMC6476855

[brb33507-bib-0035] Lowry, O. , Rosebrough, N. , Farr, A. L. , & Randall, R. (1951). Protein measurement with Folin‐phenol reagent. Journal of Biological Chemistry, 193(1), 265–275. 10.1016/S0021-9258(19)52451-6 14907713

[brb33507-bib-0036] Lynch, M. A. (2015). Neuroinflammatory changes negatively impact on LTP: A focus on IL‐1β. Brain Research, 1621, 197–204. 10.1016/j.brainres.2014.08.040 25193603

[brb33507-bib-0037] Ma, L. , Seager, M. A. , Wittmann, M. , Jacobson, M. , Bickel, D. , Burno, M. , Jones, K. , Graufelds, V. K. , Xu, G. , Pearson, M. , Mccampbell, A. , Gaspar, R. , Shughrue, P. , Danziger, A. , Regan, C. , Flick, R. , Pascarella, D. , Garson, S. , Doran, S. , … Ray, W. J. (2009). Selective activation of the M1 muscarinic acetylcholine receptor achieved by allosteric. Proceedings of the National Academy of Sciences of the United States of America, 106(37), 15950–15955. 10.1073/pnas.0900903106 19717450 PMC2732705

[brb33507-bib-0038] Mohammadpour, T. , Hosseini, M. , Naderi, A. , Karami, R. , Sadeghnia, H. R. , Soukhtanloo, M. , & Vafaee, F. (2015). Protection against brain tissues oxidative damage as a possible mechanism for the beneficial effects of *Rosa damascena* hydroalcoholic extract on scopolamine induced memory impairment in rats. Nutritional Neuroscience, 18(7), 329–336. 10.1179/1476830514Y.0000000137 24974980

[brb33507-bib-0039] Naquvi, K. J. , Ansari, S. , Ali, M. , & Najmi, A. (2014). Volatile oil composition of *Rosa damascena* Mill. (Rosaceae). Journal of Pharmacognosy and Phytochemistry, 2, 177–181.

[brb33507-bib-0040] Nathan, P. J. , Watson, J. , Lund, J. , Davies, C. H. , Peters, G. , Dodds, C. M. , Swirski, B. , Lawrence, P. , Bentley, G. D. , O'neill, B. V. , Robertson, J. , Watson, S. , Jones, G. A. , Maruff, P. , Croft, R. J. , Laruelle, M. , & Bullmore, E. T. (2013). The potent M1 receptor allosteric agonist GSK1034702 improves episodic memory in humans in the nicotine abstinence model of cognitive dysfunction. International Journal of Neuropsychopharmacology, 16, 721. 10.1017/S1461145712000752 22932339

[brb33507-bib-0041] Navarria, A. , Wohleb, E. S. , Voleti, B. , Ota, K. T. , Dutheil, S. , Lepack, A. E. , Dwyer, J. M. , Fuchikami, M. , Becker, A. , Drago, F. , & Duman, R. S. (2015). Rapid antidepressant actions of scopolamine: Role of medial prefrontal cortex and M1‐subtype muscarinic acetylcholine receptors. Neurobiology of Disease, 82, 254–261. 10.1016/j.nbd.2015.06.012 26102021 PMC4640941

[brb33507-bib-0042] Okano, H. , Hirano, T. , & Balaban, E. (2000). Learning and memory. Proceedings of the National Academy of Sciences of the United States of America, 97, 12403–12404. 10.1073/pnas.210381897 11035781 PMC34060

[brb33507-bib-0043] Pandareesh, M. D. , Anand, T. , & Khanum, F. (2016). Cognition enhancing and neuromodulatory propensity of *Bacopa monniera* extract against scopolamine induced cognitive impairments in rat hippocampus. Neurochemical Research, 41, 985–999. 10.1007/s11064-015-1780-1 26677075

[brb33507-bib-0044] Paxinos, G. , & Watson, C. (2007). The rat brain in stereotaxic coordinates (6th ed.). Academic Press. 10.1016/0165-0270(80)90021-7 6110810

[brb33507-bib-0045] Pellati, F. , Orlandini, G. , Van Leeuwen, K. A. , Anesin, G. , Bertelli, D. , Paolini, M. , Benvenuti, S. , & Camin, F. (2013). Gas chromatography combined with mass spectrometry, flame ionization detection and elemental analyzer/isotope ratio mass spectrometry for characterizing and detecting the authenticity of commercial essential oils of *Rosa damascena* Mill. Rapid Communications in Mass Spectrometry, 27(5), 591–602. 10.1002/rcm.6489 23413218

[brb33507-bib-0046] Ramezani, R. , Moghimi, A. , Rakhshande, H. , Ejtehadi, H. , & Kheirabadi, M. (2008). The effect of *Rosa damascena* essential oil on the amygdala electrical kindling seizures in rat. Pakistan Journal of Biological Sciences, 11(5), 746–751. 10.3923/pjbs.2008.746.751 18819571

[brb33507-bib-0047] Rezvani‐Kamran, A. , Salehi, I. , Shahidi, S. , Zarei, M. , Moradkhani, S. , & Komaki, A. (2017). Effects of the hydroalcoholic extract of *Rosa damascena* on learning and memory in male rats consuming a high‐fat diet. Pharmaceutical Biology, 55(1), 2065–2073. 10.1080/13880209.2017.1362010 28832226 PMC6130717

[brb33507-bib-0048] Sajjad Haider, M. , Ashraf, W. , Javaid, S. , Rasool, M. F. , Rahman, H. M. A. , Saleem, H. , Anjum, S. M. M. , Siddique, F. , Morales‐Bayuelo, A. , Kaya, S. , & Alqahtani, F. (2021). Chemical characterization and evaluation of the neuroprotective potential of *Indigofera sessiliflora* through in‐silico studies and behavioral tests in scopolamine‐induced memory compromised rats. Saudi Journal of Biological Sciences, 28(8), 4384–4398. 34354423 10.1016/j.sjbs.2021.04.033 PMC8325032

[brb33507-bib-0049] Scarpa, M. , Hesse, S. , & Bradley, S. J. (2020). M1 muscarinic acetylcholine receptors: A therapeutic strategy for symptomatic and disease‐modifying effects in Alzheimer's disease? Advances in Pharmacology, 88, 277–310. 10.1016/bs.apha.2019.12.003 32416870

[brb33507-bib-0050] Schellhammer, I. , & Rarey, M. (2007). TrixX: Structure‐based molecule indexing for large‐scale virtual screening in sublinear time. Journal of Computer‐Aided Molecular Design, 21(5), 223–238. 10.1007/s10822-007-9103-5 17294247

[brb33507-bib-0051] Schliebs, R. , & Arendt, T. (2011). The cholinergic system in aging and neuronal degeneration. Behavioural Brain Research, 221(2), 555–563. 10.1016/j.bbr.2010.11.058 21145918

[brb33507-bib-0052] Senol, F. , Orhan, I. , Kürkçüoğlu, M. , Khan, M. , Altintas, A. , Şener, B. , & Başer, K. (2011a). An in vitro approach to neuroprotective activity of *Rosa damascena* Mill, a medieval age traditional medicine used for memory enhancement. Planta Medica, 77(12), 1440–1440. 10.1055/s-0031-1282921

[brb33507-bib-0053] Senol, F. S. , Orhan, I. E. , Kurkcuoglu, M. , Khan, M. T. H. , Altintas, A. , Sener, B. , & Baser, K. H. C. (2011b). A mechanistic investigation on anticholinesterase and antioxidant effects of rose (*Rosa damascena* Mill.). Food Research International, 53(1), 502–509. 10.1016/j.foodres.2013.05.031

[brb33507-bib-0054] Sharma, B. , Singh, N. , & Singh, M. (2008). Modulation of celecoxib‐ and streptozotocin‐induced experimental dementia of Alzheimer's disease by pitavastatin and donepezil. Journal of Psychopharmacology, 22(2), 162–171. 10.1177/0269881107081553 18208924

[brb33507-bib-0055] Spencer, J. P. E. (2010). The impact of fruit flavonoids on memory and cognition. British Journal of Nutrition, 104(3), S40–S47. 10.1017/S0007114510003934 20955649

[brb33507-bib-0056] Takamori, M. (2019). Synaptic compensatory mechanism and its impairment in autoimmune myasthenic diseases. Journal of Immunological Sciences, 3(3), 6–13. 10.29245/2578-3009/2019/3.1173

[brb33507-bib-0057] Tomàs, J. , Garcia, N. , Lanuza, M. A. , Santafé, M. M. , Tomàs, M. , Nadal, L. , Hurtado, E. , Simó, A. , & Cilleros, V. (2017). Presynaptic membrane receptors modulate ACh release, axonal competition and synapse elimination during neuromuscular junction development. Frontiers in Molecular Neuroscience, 10, 132. 10.3389/fnmol.2017.00132 28559796 PMC5432534

[brb33507-bib-0058] Tong, L. , Prieto, G. A. , Kramár, E. A. , Smith, E. D. , Cribbs, D. H. , Lynch, G. , & Cotman, C. W. (2012). Brain‐derived neurotrophic factor‐dependent synaptic plasticity is suppressed by interleukin‐1β via p38 mitogen‐activated protein kinase. Journal of Neuroscience, 32(49), 17714–17724. 10.1523/JNEUROSCI.1253-12.2012 23223292 PMC3687587

[brb33507-bib-0059] UniProt Consortium . (2021). UniProt: The Universal Protein Knowledgebase in 2021. Nucleic Acids Research, 49(D1), D480–D489. 10.1093/nar/gkaa1100 33237286 PMC7778908

[brb33507-bib-0060] Uslaner, J. M. , Eddins, D. , Puri, V. , Cannon, C. E. , Sutcliffe, J. , Chew, C. S. , Pearson, M. , Vivian, J. A. , Chang, R. K. , Ray, W. J. , Kuduk, S. D. , & Wittmann, M. (2013). The muscarinic M1 receptor positive allosteric modulator PQCA improves cognitive measures in rat, cynomolgus macaque, and rhesus macaque. Psychopharmacology, 225(1), 21–30. 10.1007/s00213-012-2788-8 22825578

[brb33507-bib-0061] Verma, S. , Padalia, C. , & Chauhan, A. (2011). Chemical investigation of the volatile components of shade‐dried petals of damask rose (*Rosa damascena* Mill.). Archives of Biological Sciences, 63(4), 1111–1115. 10.2298/ABS1104111V

[brb33507-bib-0062] Volkamer, A. , Griewel, A. , Grombacher, T. , & Rarey, M. (2010). Analyzing the topology of active sites: On the prediction of pockets and subpockets. Journal of Chemical Information and Modeling, 50(11), 2041–2052. 10.1021/ci100241y 20945875

[brb33507-bib-0063] Vorhees, C. V. , & Williams, M. T. (2006). Morris water maze: Procedures for assessing spatial and related forms of learning and memory. Nature Protocols, 1(2), 848–858. 10.1038/nprot.2006.116 17406317 PMC2895266

[brb33507-bib-0064] Wang, Q. , Sun, L.‐H. , Jia, W. , Liu, X.‐M. , Dang, H.‐X. , Mai, W.‐L. , Wang, N. , Steinmetz, A. , Wang, Y.‐Q. , & Xu, C.‐J. (2010). Comparison of ginsenosides Rg1 and Rb1 for their effects on improving scopolamine‐induced learning and memory impairment in mice. Phytotherapy Research, 24(12), 1748–1754. 10.1002/ptr.3130 20564503

[brb33507-bib-0065] Waterhouse, A. , Bertoni, M. , Bienert, S. , Studer, G. , Tauriello, G. , Gumienny, R. , Heer, F. T. , De Beer, T. A. P. , Rempfer, C. , Bordoli, L. , Lepore, R. , & Schwede, T. (2018). SWISS‐MODEL: Homology modelling of protein structures and complexes. Nucleic Acids Research, 46(W1), W296–W303. 10.1093/nar/gky427 29788355 PMC6030848

[brb33507-bib-0066] Wohleb, E. S. , Wu, M. , Gerhard, D. M. , Taylor, S. R. , Picciotto, M. R. , Alreja, M. , & Duman, R. S. (2016). GABA interneurons mediate the rapid antidepressant‐like effects of scopolamine. Journal of Clinical Investigation, 126(7), 2482–2494. 10.1172/JCI85033 27270172 PMC4922686

[brb33507-bib-0067] Zhu, S. , Li, H. , Dong, J. , Yang, W. , Liu, T. , Wang, Y. , Wang, X. , Wang, M. , & Zhi, D. (2017). Rose essential oil delayed Alzheimer's disease‐like symptoms by SKN‐1 pathway in *C. elegans* . Journal of Agricultural and Food Chemistry, 65(40), 8855–8865. 10.1021/acs.jafc.7b03224 28915354

