# Supplementary material for: A comprehensive assessment of the cholinergic‐supporting and cognitive‐enhancing effects of Rosa damascena Mill. (Damask rose) essential oil on scopolamine‐induced amnestic rats
Source: Brain Behav. 2024 Apr 30;14(5):e3507. doi: 10.1002/brb3.3507 (PMC11061205; doi:10.1002/brb3.3507)
Supplement: Supplementary file 1 — Supporting Information [file BRB3-14-e3507-s001.docx]

**SUPPORTING INFORMATION**

**A comprehensive assessment of the cholinergic-supporting and cognitive-enhancing effects of *Rosa damascena* Mill. (Damask rose) essential oil on scopolamine-induced amnestic rats**

Kerem Teralı^1,*^, Dilek Ozbeyli^2^, Duygu Yiğit-Hanoğlu^3^, Kemal Hüsnü Can Başer^3^, Göksel Şener^4^, Asli Aykac^5^

1. Department of Medical Biochemistry, Faculty of Medicine, Cyprus International University, Nicosia, 99258, Cyprus
2. Department of Medical Services and Techniques, Vocational School of Health Services, Marmara University, Istanbul, 34865, Turkey
3. Department of Pharmacognosy, Faculty of Pharmacy, Near East University, Nicosia, 99138, Cyprus
4. Department of Pharmacology, Fenerbahce University, Istanbul, 34758, Turkey
5. Department of Biophysics, Near East University, Nicosia, 99138, Cyprus

^*^ Corresponding author

**SUPPLEMENTARY METHODS**

**Analysis and identification of *R. damascena* essential oil**

The commercial essential oil of *R. damascena* Mill. (RDEO) was acquired from Sebat, Inc. (Isparta, Turkey), with the identification of the plant source material being performed by Prof. K. H. C. Baser. After RDEO was diluted 1:2 (v/v) with *n*-hexane, gas chromatography/mass spectrometry (GC/MS) analyses were conducted. For gas chromatography/flame ionization detection (GC/FID) analyses, it was used at a 1:1 dilution ratio (*i.e.* as an undiluted stock solution). An Agilent 6890N GC system was used in the GC analysis. RDEO was examined by capillary GC and GC/MS simultaneously using an Agilent GC–MSD system (5975; Agilent Technologies Inc., Santa Clara, CA). INNOWax Agilent GC column (HP, USA) (60 m × 0.25 mm; film thickness 0.25 mm) was used for the analysis of simultaneous injections and the FID temperature was set at 300 °C. He^2+^ was used as the carrier gas, with a flow rate of 0.8 ml/min. GC oven was held constant at 60 °C for 10 min before increasing to 220 °C at a rate of 4 °C/min and then regulated to 240 °C at a rate of 1 °C/min. The split ratio was adjusted to 40:1. The injector temperature was set at 250 °C. At 70 eV, mass spectra were collected. The mass range was *m*/*z* 35–450. To identify the RDEO components, the relative retention indices were compared with the *n*-alkane series (Pellati *et al.*, 2013).

**SUPPLEMENTARY TABLES**

**Supplementary Table 1.** Components of *R. damascena* essential oil as identified by content analysis.

| **RRI** | | **Compound** | | **Relative percentage amounts (%) of the separated compounds in RDEO** | | |  |
| --- | --- | --- | --- | --- | --- | --- | --- |
| 1021 | | α-Pinene | | | 0.6 | |  |
| 1119 | | β-Pinene | | | 0.1 | |  |
| 1132 | | Sabinene | | | 0.1 | |  |
| 1172 | | Myrcene | | | 0.1 | |  |
| 1262 | | γ-Terpinene | | | 0.1 | |  |
| 1361 | | 1-Hexanol | | | 0.1 | |  |
| 1368 | | *cis*-Rose oxide | | | 0.1 | |  |
| 1501 | | Pentadecane | | | 0.4 | |  |
| 1615 | | α-Guaiene | | | 0.1 | |  |
| 1624 | | Terpinene-4-ol | | | 0.3 | |  |
| 1629 | | β-Caryophyllene | | | 0.2 | |  |
| 1675 | | Citronellyl acetate | | | 0.2 | |  |
| 1702 | | Heptadecane | | | 2.6 | |  |
| 1704 | | α-Humulene | | | 0.1 | |  |
| 1717 | | α-Terpineol | | | 0.2 | |  |
| 1727 | | Heptadecene | | | 0.1 | |  |
| 1741 | | Neryl acetate | | | 0.1 | |  |
| 1744 | | Germacrene D | | | 0.5 | |  |
| 1747 | | δ-Guaiene | | | 0.1 | |  |
| 1757 | | Geranial | | | 0.5 | |  |
| 1771 | | Geranyl acetate | | | 0.6 | |  |
| 1777 | | Citronellol | | | 29.5 | |  |
| 1802 | | Octadecane | | | 0.3 | |  |
| 1816 | | Nerol | | | 9.6 | |  |
| 1826 | | *iso*-Geraniol | | | 0.2 | |  |
| 1847 | | 2-Phenylethyl formate | | | 0.3 | |  |
| 1860 | | Geraniol | | | 20.2 | |  |
| 1903 | | Nonadecane | | | 16.5 | |  |
| 1923 | | Nonadecene | | | 3.2 | |  |
| 1944 | | Phenylethyl alcohol | | | 1.1 | |  |
| 2001 | | Eicosane | | | 1.4 | |  |
| 2022 | | Eicosene | | | 0.3 | |  |
| 2037 | | Methyl eugenol | | | 1.6 | |  |
| 2102 | | Heneicosane | | | 5.5 | |  |
| 2121 | | 1-Tridecanol | | | 0.1 | |  |
| 2141 | | Tetracosene | | | 0.1 | |  |
| 2206 | | Eugenol | | | 0.5 | |  |
| 2302 | | Tricosane | | | 0.9 | |  |
| 2370 | | (*2E*,*6E*)-Farnesol | | | 0.9 | |  |
|  |  | | **Total** | | | 99.4 | |

*RRI: relative retention indices (versus *n*-alkanes in a polar column)

**SUPPLEMENTARY REFERENCES**

Pellati F, Orlandini G, van Leeuwen KA, Anesin G, Bertelli D, Paolini M, Benvenuti S, Camin F. Gas chromatography combined with mass spectrometry, flame ionization detection and elemental analyzer/isotope ratio mass spectrometry for characterizing and detecting the authenticity of commercial essential oils of *Rosa damascena* Mill. *Rapid Commun Mass Spectrom*. 2013;27(5):591-602.
